# A Novel and Rapid Serum Detection Technology for Non-Invasive Screening of Gastric Cancer Based on Raman Spectroscopy Combined With Different Machine Learning Methods

**DOI:** 10.3389/fonc.2021.665176

**Published:** 2021-09-27

**Authors:** Mengya Li, Haiyan He, Guorong Huang, Bo Lin, Huiyan Tian, Ke Xia, Changjing Yuan, Xinyu Zhan, Yang Zhang, Weiling Fu

**Affiliations:** ^1^ Department of Laboratory Medicine, First Affiliated Hospital, Third Military Medical University (Army Medical University), Chongqing, China; ^2^ Department of Laboratory Medicine, Chongqing University Cancer Hospital, Chongqing, China

**Keywords:** Raman spectroscopy, gastric cancer, machine learning, random forest, convolutional neural network

## Abstract

Gastric cancer (GC) is the fifth most common cancer in the world and a serious threat to human health. Due to its high morbidity and mortality, a simple, rapid and accurate early screening method for GC is urgently needed. In this study, the potential of Raman spectroscopy combined with different machine learning methods was explored to distinguish serum samples from GC patients and healthy controls. Serum Raman spectra were collected from 109 patients with GC (including 35 in stage I, 14 in stage II, 35 in stage III, and 25 in stage IV) and 104 healthy volunteers matched for age, presenting for a routine physical examination. We analyzed the difference in serum metabolism between GC patients and healthy people through a comparative study of the average Raman spectra of the two groups. Four machine learning methods, one-dimensional convolutional neural network, random forest, support vector machine, and K-nearest neighbor were used to explore identifying two sets of Raman spectral data. The classification model was established by using 70% of the data as a training set and 30% as a test set. Using unseen data to test the model, the RF model yielded an accuracy of 92.8%, and the sensitivity and specificity were 94.7% and 90.8%. The performance of the RF model was further confirmed by the receiver operating characteristic (ROC) curve, with an area under the curve (AUC) of 0.9199. This exploratory work shows that serum Raman spectroscopy combined with RF has great potential in the machine-assisted classification of GC, and is expected to provide a non-destructive and convenient technology for the screening of GC patients.

## Introduction

Gastric cancer (GC) is a clinically common malignant tumor of the digestive tract, accounting for 5.7% of the total new incidences of malignant tumors (1). Its mortality rate is about the second highest among malignant tumor diseases, and the high incidence of GC worldwide is concentrated in developing countries, especially China (2). Because the early symptoms of GC are not specific enough, people tend to ignore or misjudge the condition, leading to some patients whose condition has progressed to the middle and late stages when they are diagnosed. Thus, the optimal treatment time is missed, and the treatment effect and prognosis are relatively poor (3). Therefore, improving the diagnosis rate detection rate of early GC is of significance for reducing the mortality of GC. At present, the clinical diagnosis of GC mainly uses CT and gastrointestinal endoscopic biopsy techniques. However, during CT examinations, breathing artifacts are prone to occur, affecting the diagnosis and treatment results (4). Although gastroscopic biopsy, which is the gold standard for GC diagnosis, has reliable accuracy, it is difficult to popularize it to routine screening diagnosis because gastroscopy is invasive and affected by patient compliance and operator techniques. Therefore, there is an urgent need for simple and practical serum detection technology in order to help accurately identify GC patients.

Raman spectroscopy is a powerful spectroscopic technique to assess the chemical composition of samples, which is based on inelastic scattering generated by the rotational and vibrational modes of molecular bonds (5). Therefore, the spectral distributions produced by the Raman active functional groups of biomolecules with distinct chemical and molecular features (proteins, nucleic acids, lipids etc.) are different, and this can be used as “fingerprints” of compounds and mixtures. Meanwhile, changes in these “fingerprints” can provide disease information, which plays an extremely important role in disease diagnosis and monitoring of disease progress (6). With its unique technical advantages of non-destructive testing, high sensitivity, simplicity and speediness, Raman spectroscopy has shown good application potential in the fields of biomacromolecule detection, pathogenic microorganism detection, tumor disease diagnosis and other fields (7–9).

Biological fluids (such as blood, urine, saliva, etc.) contain a variety of chemical components, reflecting the metabolism of the body. Because of their advantages such as easy collection, low risk of invasion, and repeatable sampling, biological fluids have been widely applied in clinical. In recent years, label-free Raman spectroscopy of biological fluids combined with machine learning methods has been extensively exploited for early disease screening and cancer staging research.

Because the scattering cross section of some molecules is very small, the Raman scattering signal is weak and easily interfered by the fluorescent background, resulting in the insignificant difference in the spectra of normal and diseased serum samples (10). Therefore, advanced statistical analysis techniques are needed to extract effective information from the enormous Raman spectra datasets to distinguish these samples. K-nearest neighbor (KNN), a relatively mature, comprehensible and simple machine learning method, has always been the classic algorithm in popularity. Support vector machine (SVM) is a powerful and supervised machine learning algorithm. Employing kernel functions, SVM transforms the original data to a higher dimensional feature space, where the data might be linearly separable. At the same time, a decision boundary (called hyperplane) is created so that the two classes can be separated correctly and the classification interval is maximum (11). Random forest (RF) is a kind of ensemble learning algorithm with fast training speed and high model robustness, which was proposed by Breiman in 2001 (12). RF uses bootstrap sampling method to extract multiple samples from the original data set. Each bootstrap sample participates in the construction of a decision tree, and the prediction results of the final model are determined by voting on the classification results of multiple decision trees (13). By building a large number of decision trees, RF has the advantages of anti-noise, preventing over-fitting, and strong predictive ability, and has been increasingly applied in various types of data mining. Deep learning is one of the hottest areas of research in recent years, and its development has created great opportunities in the fields of chemistry and biology. Convolutional Neural Network (CNN) is one of the representative deep learning algorithms for high-dimensional data, which is constructed by imitating biological visual perception mechanism. As an advanced technology with strong learning ability, CNN has excellent performance in Raman spectral analysis. For example, Wang et al. could assess the biochemical signatures of different *Arcobacter* strains by using Raman spectroscopy combined with CNN, and achieved a recognition accuracy of 97.2% for 18 *Arcobacter* species (14). Shao et al. used CNN model to identify the serum Raman spectrum of prostate cancer patients with bone metastases, and obtained a testing classification accuracy of 81.7% (15). In addition, Hollon et al. completed a significant study that combined stimulated Raman histology with CNN to automate the diagnosis of intraoperative brain tumors in near real-time, the diagnostic results can be predicted within 150 seconds with an overall accuracy of 94.6% (16). These studies indicate the great potential of combining deep learning and Raman spectroscopy for classification.

In this study, we analyzed the differences in serum Raman spectra of GC patients and normal subjects, and explored the metabolic differences between them. Four promising machine learning methods (1D-CNN, RF, SVM and KNN) were developed for discriminant analysis of two groups of Raman spectral data, and the actual performance of these four methods was evaluated. This work may provide a non-destructive, fast and simple serum test for screening of GC.

## Methods

### Collection and Preparation of Serum Samples

Serum samples of 109 patients with GC were collected in the First Affiliated Hospital of Army Medical University from May 2019 to January 2020, including 35 cases in stage I, 14 cases in stage II, 35 cases in stage III, and 25 cases in stage IV. All patients need to meet the following conditions: diagnosed with GC by gastroscopy plus pathological biopsy; no tumors of other systemic systems; no serious dysfunction of heart, lung, liver, kidney and other organs; no surgery or chemotherapy prior to sample collection. In the meantime, serum samples of 104 healthy volunteers matching the age of the GC group from the physical examination department were collected as the control group. All the included healthy controls had no history of gastrointestinal disease. Informed consent was obtained for all participants in this experiment. And this study was approved by the Ethics Committee of the First Affiliated Hospital of Army Medical University (approval no.KY2020165).

After an overnight fast for 10 hours, 3 ml of peripheral blood was collected from each subject. The blood was centrifuged at 3000 rpm for 10 min after coagulation. The supernatant serum sample was collected in a special cryopreservation tube and stored in a -80°C refrigerator until the Raman measurement was performed.

### Raman Spectra Measurements and Data Preprocessing

A Raman micro-spectrometer (XploRA PLUS, Horiba Scientifics, France) was used to acquire serum Raman spectra at excitation wavelength of 532 nm and power of 6.3 mW. The laser was focused on the dried serum sample through a 100x magnification objectives, and the Raman spectrum in the range of 600-1800 cm^-1^ was recorded. The total acquisition time of each spectrum is 20 s. Every sample was measured five times in different spots, and the average spectrum was taken for further analysis

**Figure 1:**
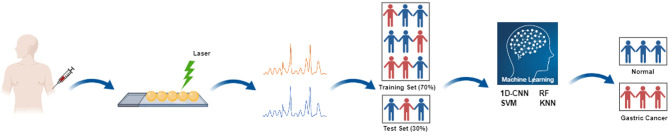
Flow chart of serum Raman spectroscopy combined with different machine learning methods for non-invasive screening of gastric cancer. Four popular and high-performance machine learning methods include one-dimensional convolutional neural network (1D-CNN), random forest (RF), support vector machine (SVM), and k-nearest neighbor (KNN).

Prior to data analysis, LabSpec 6 software (Horiba Scientifics, France) was used to preprocess Raman spectra, including smoothing, baseline correction and normalization, to filter the interference noise and remove the fluorescence background. The processed data was analyzed using different machine learning methods, including 1D-CNN, RF, SVM and KNN. The specific experimental flow chart is shown in **Figure 1**.

### 1D - CNN

**Figure 2:**
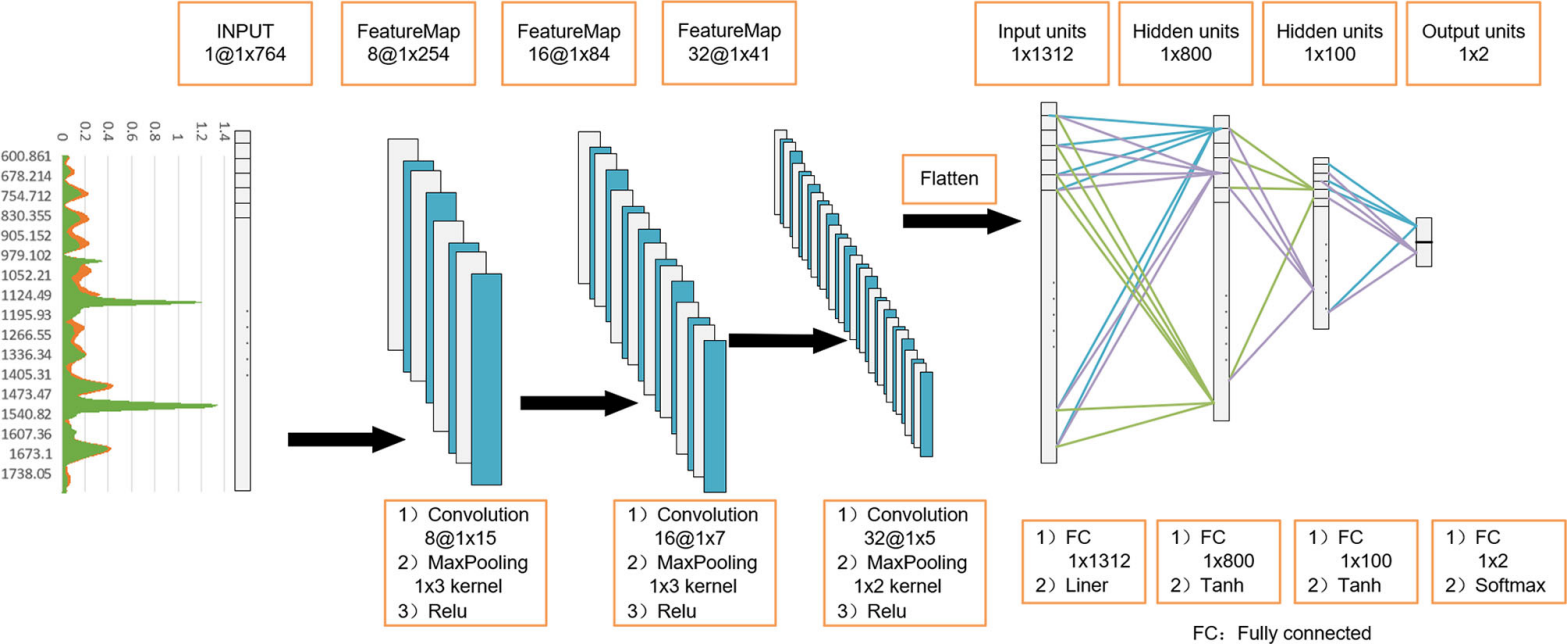
One-dimensional convolutional neural network (1D-CNN) model architecture based on serum Raman spectra. Using 3-layer 1D convolution block, combined with fully connected neural network, construct a gastric cancer serum Raman spectrum discrimination model.

Since Raman spectrum signals are one-dimensional, referring to the classic model structure of LeNet-5, we developed a 1D-CNN model to identify the serum Raman spectrum of GC. Its model structure can be viewed in **Figure 2**. In the model, one input layer, three convolutional layers, three fully connected layers, and a Softmax output layer are included. The input data is 764 nodes. In the convolution layer, there are one convolution function, one activation function and one pooling function. And the convolution kernel size of each layer is set as 15x1, 7x1 and 5x1 respectively. After dimension reduction by the Max-Pooling method on each layer, 32 Raman features are obtained. These Raman features were flattened into a one-dimensional vector, connected to the full connection layer with the number of neurons of 1312,800,100, and finally connected to the last output layer. The number of neurons in the output layer was set to 2, representing negative (normal group) and positive (GC group). In this 1D-CNN model, the learning rate was 0.0005.

The accuracy-epoch and loss-epoch curves of the 1D-CNN model were calculated, and the results are shown in **Figure S1**. As the epoch increased, whether it was the training set or the test set, the accuracy-epoch curves first raised quickly and then gradually reached a plateau (**Figure S1A**). As shown in **Figure S1B**, the loss-epoch curves had a downward trend in both the training set and the test set. This indicates that the training process of the 1D-CNN model is effective.

### RF

**Figure 3:**
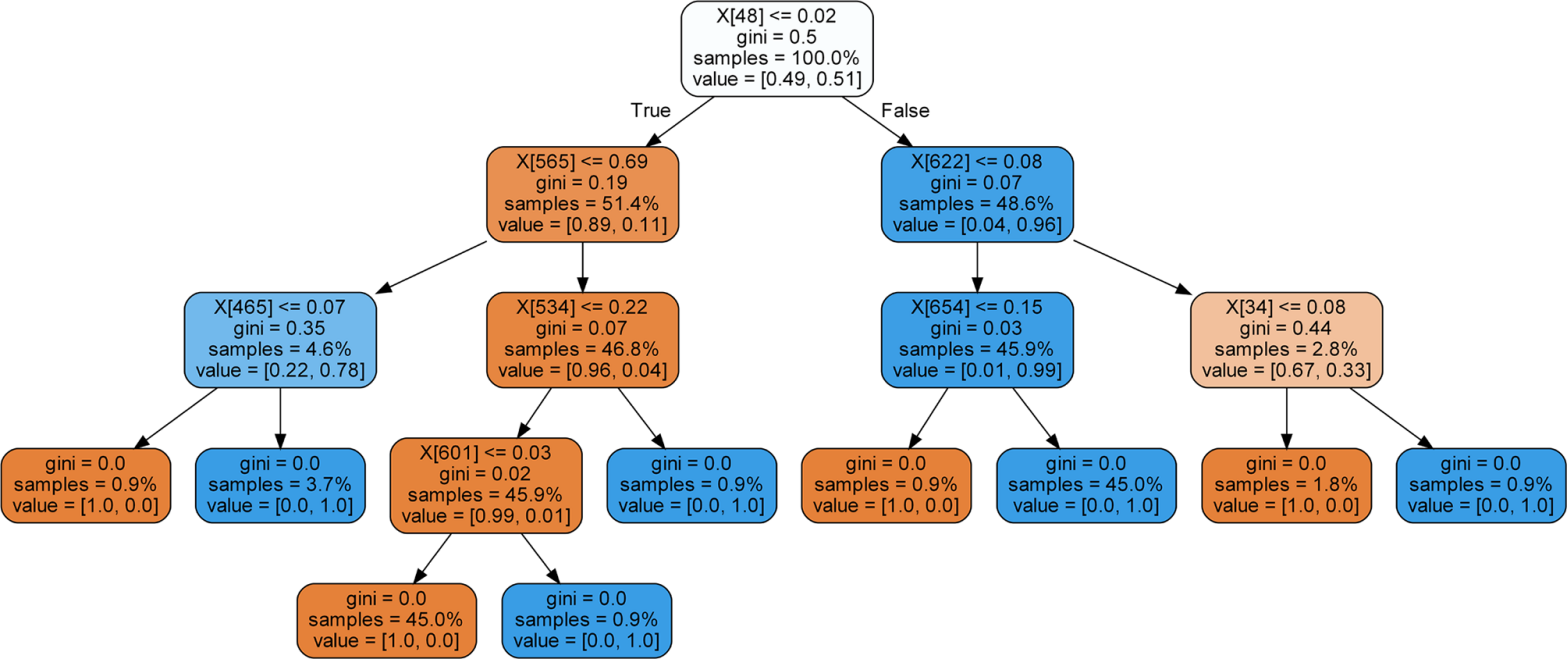
Single decision tree structure diagram of random forest (RF) model.

Given the Raman spectroscopy dataset contains N samples, which is defined as *L_N_* = {*l*_1_, *l*_2_, *l*_3_,…, *l_n_*} ∈ *R^N^* ^×^ *^K^*. Each sample in it has K-dimension frequency response features, which defines the attribute dataset as *A* = {*a* ^1^, *a* ^2^, *a* ^3^,…, *a^K^*}. There are two possible values *V* = {*Positive*, *Negative*} for every attribute, which directs over the threshold and below the threshold, respectively. As the RF is a special bagging method of decision trees, the Raman spectroscopy RF classification model in this manuscript adopted classical CART decision trees (17). For each node in CART decision tree, to get the best optimization dividing of subtree, the Gini Index is instructed to calculate the information gain rate of every decision node. Firstly, the calculation of Gini is defined as:


$$\text{Gini}\left(L\right)=\sum_{n=1}^{N}\sum_{n^{\prime }\neq n}p_{n}p_{n^{\prime }}=1-\sum_{n=1}^{N}p_{n}^{2}$$

where the *p_n_* is the probability of the evaluation samples belong to class n. Therefore, the smaller result of *Gini*(*L)*, the more purity of the decision node. As the feature of Raman spectroscopy is composed by a K-dimension frequency response, the Gini Index which is used for optimizing the dividing of subtree is defined below:


$$\text{Gini\_index}\left(L,a\right)=\sum_{v=1}^{V}\frac{\left|L^{v}\right|}{\left|L\right|}\text{Gini}\left(L^{v}\right)$$

where the *L^v^* is represent the number of samples which the attribute gets a value of v. The best optimization dividing of subtree is the attribute has the smallest result of Gini Index. It can convert to the object function:


$$a_{*}=\text{argmin Gini\_index}\left(L\text{, }a\right)$$

where the a_*_ is the attribute makes the Gini Index smallest. Especially, when the output of Gini Index is zero, the current evaluation node is set to the final leaf node which exports the classification result. In our RF classification model, there were *m* decision trees. We have trained all the decision trees by the construction standard described above. The final output of the RF classification model was the combination prediction results of all the decision trees by majority voting algorithm. The visualization of a single decision tree in our model is shown in **Figure 3**. In the RF model, the number of decision trees constructed was set as 300, and the maximum leaf node depth was set as 200.

### SVM

In this paper, SVM based on Radial Basis Function (RBF) was used for experiments. There are two main parameters, the penalty coefficient C and the gamma value of the RBF kernel function. C is used to punish the tolerance of classification error in training. Increasing the C value can make the model better fit the classification hyperplane of training samples, but it is easy to cause overfitting. On the contrary, if the C value is reduced, it is easy to cause underfitting. The gamma value determines the data mapping to the new feature space. The larger the gamma value, the less support vectors calculated by the model for classification will be. In the experiment, a cross-validation evaluation method was used to optimize parameter C and gamma. For the Raman spectroscopy prediction task, the optimal C was 5 and gamma was 0.5.

### KNN

As a classic and simple supervised recognition method, KNN was used for comparative research on effectiveness. In this paper, KNN adopted the 3-nearest neighbor model. The distance of reference point of sample classification was measured based on Euclidean space.

### Model Training and Test Performance Evaluation

In this study, for all training and testing procedures, the four machine learning methods were implemented using Pytorch and Sklearn. At the same time, to ensure all the data including the GC group and the normal group are randomly split into training set and test set, the Dataloader class in Pytorch was imported and the shuffle attribute was set to true. Since the sample sizes of the gastric cancer group and the normal group are similar, randomization can ensure the similar data distribution and proportions in the process of division.

In order to test the validity and robustness of these classification models, the data set was divided into two subsets. The training set contains 70% samples of total dataset, and the testing set consists of the left 30% samples. All the samples in each set were selected by random sampling. In addition, for the purpose of exploring the 1D-CNN model classification effectiveness on a small sample size dataset, three different proportions of training sets and test sets were constructed, including 8:2 (retaining 20% of the samples as test samples), 7:3 (retaining 30% of the samples as test samples) and 6:4 (retaining 40% of the samples as test samples). For every different division ratio of each method, 10 independent evaluation experiments were conducted. The final result was an average of the 10 evaluation experiments.

In order to evaluate the diagnostic efficiency of the four machine learning classification models, some common parameters were used, including accuracy (Acc), sensitivity (Se), specificity (Sp), positive predictive value (PPV), negative predictive value (NPV). Their calculation equations are as follows:


$$Acc=\frac{TP+TN}{P+N}\;$$

$$Se=\frac{TP}{TP+FN}$$

$$Sp=\frac{TN}{TN+FP}$$

$$PPV=\frac{\text{TP}}{\text{TP}+\text{FP}}\;$$

$$NPV=\frac{TN}{\text{TN}+\text{FN}}\;$$

Where P, N, TP, FP, FN and TN represent actual positive, actual negative, true positive, false positive, false negative and true negative, respectively.

## Results

### Baseline Characteristics

In this study, there were 109 people in the GC group and 104 people in the control group. The baseline characteristics are shown in **Table 1**. There were 82 males and 27 females in the GC group. The results are consistent with a previous study, which is a significant gender difference in the incidence of GC (18). The incidence of GC in men is significantly higher than that in women. Besides, we found there was no significant difference in age between the two groups (P =0.388).

**Table 1:** Detailed information about the subjects in this study.

	Gastric Cancer (n = 109)				Normal (n = 104)
	Stage I (n = 35)	Stage II (n = 14)	Stage III (n = 35)	Stage IV (n = 25)	
Age	55.00 ± 12.353	55.71 ± 10.194	55.77 ± 12.932	59.44 ± 9.904	55.18 ± 7.500
Gender					
Male	22 (62.9%)	12 (85.7%)	27 (77.1%)	21 (84.0%)	54 (51.9%)
Female	13 (37.1%)	2 (14.3%)	8 (22.9%)	4 (16.0%)	50 (48.1%)

### Raman Spectra Analysis

**Figure 4:**
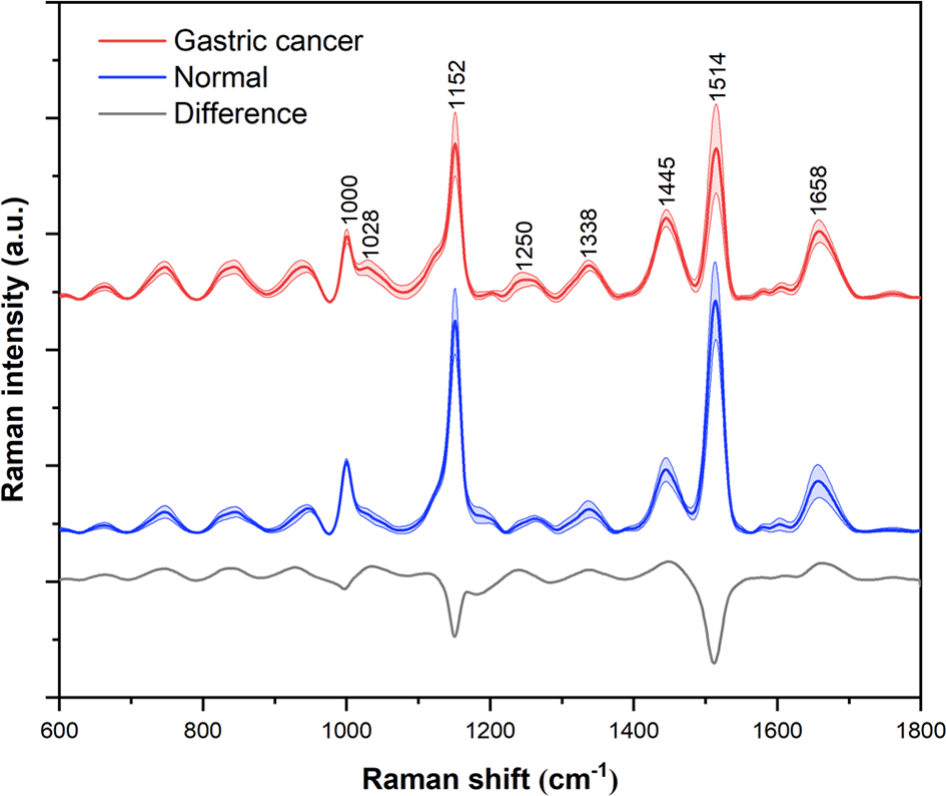
Normalized average Raman spectra of gastric cancer (red) and normal (blue) serum samples. The shaded areas represent the standard deviations. Also shown at the bottom is the difference spectrum of gastric cancer minus normal. For the purpose of clarity, spectra have been offset.

A total of 545 serum Raman spectra from GC patients and 520 serum Raman spectra from normal individuals were collected successfully. **Figure 4** shows the normalized average spectra ±1 standard deviations from the two sample groups. Stable and distinct peaks at 1000, 1152, 1445, 1514 and 1658 cm^-1^ were observed in all the Raman spectra of diseased and control group. According to the previous literatures and studies, the peak position, the vibrational mode and tentative molecular assignments of these major Raman peaks are summarized in **Table 2** (19–21). The bottom of the figure shows the difference spectrum of the normalized average spectrum of the GC group minus the normalized average spectrum of the normal group, reflecting the spectral differences between the two groups more clearly and intuitively. Compared with the normal group serum Raman spectra, the Raman peak intensity of GC serum at 1000, 1152, 1514 cm^-1^ is lower. However, the Raman peaks of GC have higher intensity at 1445 and 1658 cm^-1^.

**Table 2:** The peak position and tentative vibrational mode assignment of the serum Raman spectra.

Raman shift (cm^-1^)	Vibrational mode	Assignment
1000	C-C aromatic ring stretching	Phenylalanine
1152	C-C stretching	Carotenoid
1445	CH_2_ bending	Collagen, phospholipids
1514	C=C stretching	Carotenoid
1658	C=O stretching	Amide I (α-helix)

### Machine Learning Methods

In order to evaluate the ability of serum Raman spectroscopy to distinguish between the GC group and the normal group, exploratory analysis was performed using four machine learning classification models. In this study, 70% of the total data was randomly selected as the training set, and the remaining 30% was used as invisible data to assess the classification and prediction ability of the model. 10 trials were conducted for each method, and the final result adopted an average of these trials.

**Table 3** shows the test performance evaluation index results of four machine learning classification models, including accuracy, sensitivity, specificity, PPV and NPV. For 30% of the total data as test samples, the classification accuracy of 1D-CNN, RF, SVM and KNN are 88.6%, 92.8%, 91.5% and 88.9%, respectively. It is clear that the RF and the SVM classification models provide very promising results. Specifically, the RF model performed well in the following evaluation indicators: Se, Sp, PPV and NPV were 94.7%, 90.8%, 91.4% and 94.3%, respectively, and all evaluation indicators reaching more than 90%. The Sp and PPV of SVM classification model were 94.3% and 94.2%, respectively, which achieved a better result, but the sensitivity was sacrificed, and its Se and NPV were 88.9% and 89.1%, respectively. The sensitivity results of the 1D-CNN and KNN models are 94.7% and 92.6%, respectively.

**Table 3:** Evaluation of diagnostic efficiency of one-dimensional convolutional neural network (1D-CNN), random forest (RF), support vector machine (SVM), and K-nearest neighbor (KNN) classification models.

	1D-CNN (%)	RF (%)	SVM (%)	KNN (%)
Acc	88.6	92.8	91.5	88.9
Se	94.7	94.7	88.9	92.6
Sp	83.1	90.8	94.3	85.0
PPV	84.1	91.4	94.2	86.5
NPV	94.2	94.3	89.1	91.7

(Acc, Accuracy; Se, Sensitivity; Sp, Specificity; PPV, Positive Predictive Value; NPV, Negative Predictive Value).

**Figure 5:**
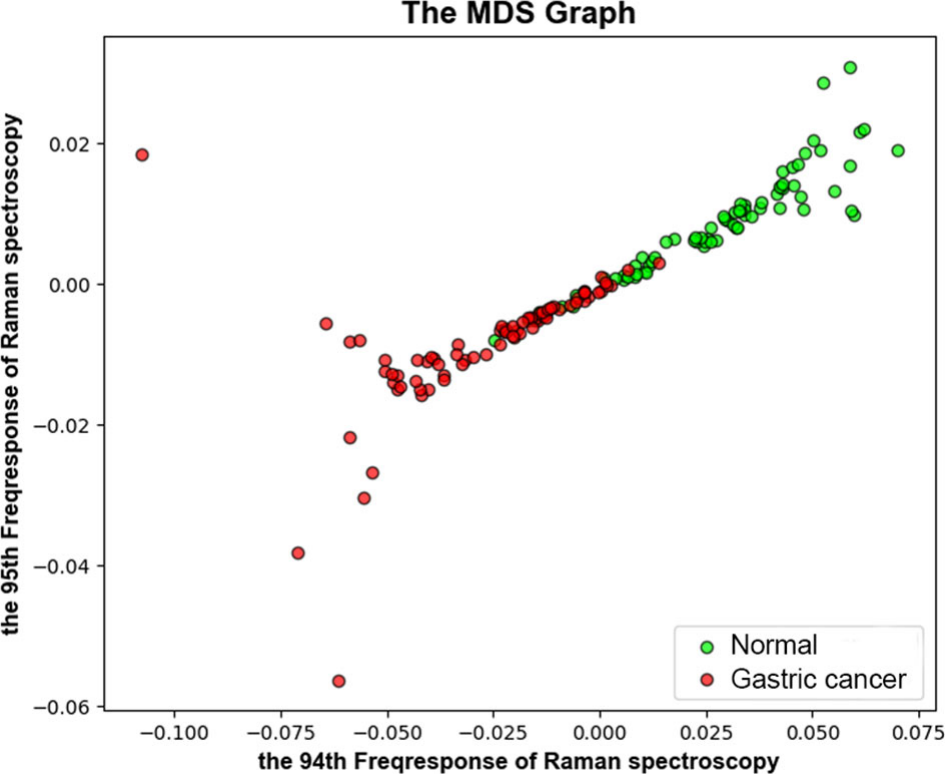
The scatter plots of the gastric cancer group and the normal group based on the spectral frequency response characteristics of the random forest (RF) classification model. Red represents the gastric cancer group, and green represents the control group.

The experiment shows that the 94th and 95th Raman spectral bands can effectively distinguish the patient with GC or not by previously modeling and training. Therefore, the two-dimensional feature space of the 94th and 95th Raman spectral bands was constructed to process data visualization and analysis in our research, which was shown in **Figure 5**. The Multidimensional Scaling (MDS) graph can clearly reveal the similarity between GC samples and normal samples in the created feature space. Each point indicates an enrolled sample, red represents the GC group and green represents the normal group. The distance between every two samples demonstrates their similarity in this feature space, the closer the distance, the higher the similarity and the further the distance, the lower the similarity. As can be seen from the MDS graph, most of the samples, come from different groups, can be effectively distinguished in this feature space (the distance between samples is far away). But a small amount of overlapping samples are displayed that it cannot robust classify all the samples only with the information of the 94th and 95th Raman spectral bands. These samples are too close, which reveals the relationship between them is similar in this feature space. In order to distinguish the overlapping samples, the remaining Raman spectral bands would be integrated together and construct the multi-dimensional feature space, as in every classification module.

**Figure 6:**
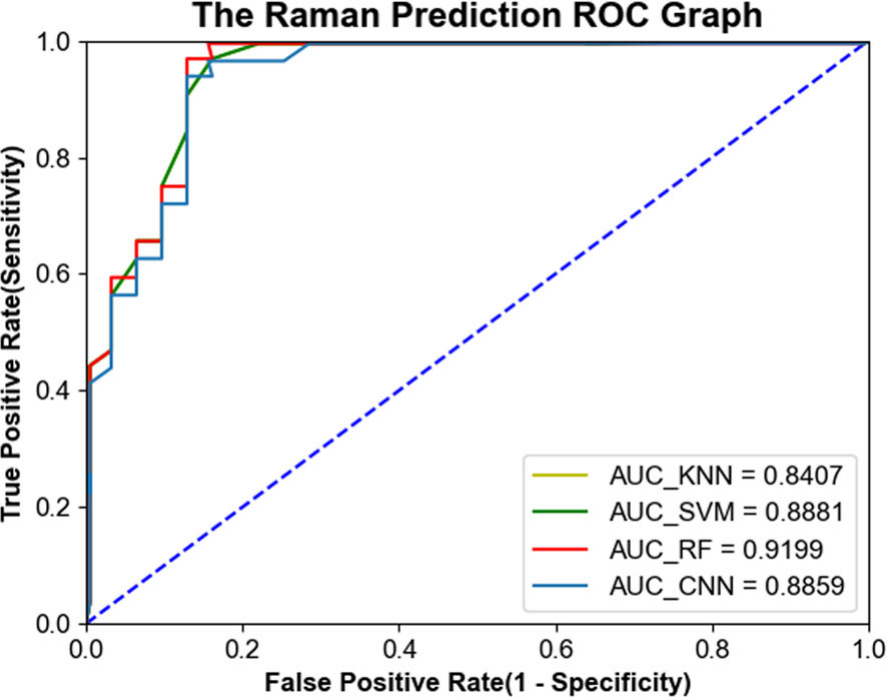
ROC curves of serum Raman spectra generated from four machine learning models, including one-dimensional convolutional neural network (1D-CNN), random forest (RF), support vector machine (SVM), and K-nearest neighbor (KNN).

In order to further evaluate the diagnostic performance of the four machine learning models, the receiver operating characteristic (ROC) curve was generated and shown in **Figure 6**. The larger the area under the curve (AUC), the better the diagnostic performance of the model. The AUC values of 1D-CNN, RF, SVM and KNN are 0.8859, 0.9199, 0.8881 and 0.8407, respectively.

The box plot **Figure S2** shows the distribution of GC prediction probability in different machine learning algorithms. Each light blue box represents the normal probability and the light yellow box indicates the GC probability. The orange line in each box shows the median value of sample distribution, and the green triangle shows the mean value of sample distribution. The whole box reveals the prediction probability range of test samples in 95% confidence intervals. And the maximum and minimum values in the distribution space are displayed at the top and bottom of each box plot, respectively. In particular, the red dots represent outlier samples in every distribution model.

As shown in **Figure S2**, the prediction probability distributions of the RF model and SVM model have large variances, which are 0.017 for the normal group and 0.016 for the GC group in the RF model; 0.018 for the normal group and 0.008 for the GC group in the SVM model. There is a wide range of test samples at 95% confidence intervals. The RF model has a 95% confidence intervals range of [0.895, 0.999] in normal prediction distribution and [0.738, 0.946] in GC. The SVM model is [0.792, 0.987] for the normal group and [0.849, 0.969] for the GC group. From the figure, it also indicates the variances of CNN are small, which are 0.004 for the normal group, 0.004 for the GC group, respectively. The 95% confidence interval range of normal and GC prediction probability are [0.907, 0.909] and [0.909, 0.910]. In addition, KNN is a special model that the classification has no probability score. So, the variances in KNN are 0, and the distribution of prediction only has one point with value 1.

In this study, 1D-CNN was further divided into training and test sets in different proportions, including 8:2 (retaining 20% of the samples as test samples), 7:3 (retaining 30% of the samples as test samples) and 6:4 (retaining 40% of the samples as test samples). **Table 4** shows the Acc, Se, Sp, PPV and NPV of the 1D-CNN model under these three sample partition ratios. When the division ratio of data training set is 0.8, 0.7 and 0.6, the average accuracy is 91.4%, 88.6% and 89.5%, respectively. This indicates that there is little correlation between test results and different proportions of data division.

**Table 4:** Test performance evaluation of one-dimensional convolutional neural network (1D-CNN) model under three division ratios.

Division ratio	Acc (%)	Se (%)	Sp (%)	PPV (%)	NPV (%)
8:2	91.4	96.1	87.4	87.2	96.4
7:3	88.6	94.7	83.1	84.1	94.2
6:4	89.5	95.5	83.6	85.7	94.9

(Training set: Test set=8:2, 7:3, 6:4).

## Discussion

As one of the most common malignant tumors, GC brings a heavy economic burden to the country and society. Therefore, early diagnosis and treatment of GC are of great significance to improve the prognosis of patients and reduce mortality. As a non-invasive method, Raman spectroscopy technology has attracted widespread attention in the field of tumor detection due to its advantages of non-destructive detection, simplicity and rapidity.

The Raman spectrum of serum is the result of different vibration modes of various biomolecules, which can reflect the changes of protein, nucleic acid and lipid in serum. Compared with normal human serum, the composition and content of biomolecules in the serum of cancer patients may have subtle changes. By analyzing the difference of the serum Raman spectra, the metabolic changes of the disease can be better understood. Our research results show that the intensity of the Raman peak at 1000 cm^-1^ attributable to phenylalanine is reduced in GC serum than in the normal group, which is consistent with the previous studies of the Raman spectrum of GC tissue (22). Moreover, the intensity of Raman peak at 1152 and 1514cm^-1^ assigned for carotenoids is also decreased. Carotenoids have antioxidant properties and may help inhibit cancer formation. Hata et al. found that the concentration of carotenoids in the skin of lesions was lower than the skin of healthy people (23). Furthermore, the Raman peak at 1445cm^-1^ caused by the CH_2_ bending mode of collagen and phospholipid is increased in GC serum. This peak has diagnostic significance as reported in previous studies of GC and lung cancer serum Raman spectroscopy (24, 25). The Raman peak at 1658 cm^-1^ belongs to the amide I band (α-helix), which is associated with the structure of the protein. Compared with normal serum, this peak is slightly increased in GC serum, which has been reported in the Raman spectrum of GC tissue (22). The metabolic disorder of tumor patients can produce the spectral results of biomolecular changes, which are different from those in normal people. But this difference is usually slight, so powerful algorithms need to be developed to diagnose diseases.

In this paper, four popular machine learning methods with high performance, including 1D-CNN, RF, SVM and KNN, were used to identify the serum Raman spectrum of GC. Our results show that the RF model has an excellent distinguishing effect for the serum Raman spectrum of GC, and the classification accuracy, sensitivity and specificity were 92.8%, 94.7% and 90.8%, respectively. Meanwhile, the ROC curve further demonstrates the excellent performance of the RF model, with an AUC value of 0.9199. The most anticipated 1D-CNN model shows the worst classification accuracy among the four algorithms, which is inconsistent with the previous excellent classification results of CNN on serum Raman spectroscopy data (15, 26). This may be because deep learning has better learning effects on high-dimensional complex data, such as high-dimension images with semantic information. Through tens of thousands to millions of massive samples to learn, the characteristics of semantic information can be obtained and expressed well. However, traditional methods are hard to extract those features well for this kind of data. Moreover, after the normalization of Raman spectral data, the representation of their features becomes more obvious, which is more suitable for traditional machine learning algorithms, such as SVM, RF, etc. Therefore, the performance results of deep learning with one-dimensional data such as Raman spectroscopy are not much different from traditional machine learning methods. Meanwhile, for deep learning, more clinical samples should be used for training to achieve better learning effect. The sample size of this paper is not large, and in the future new samples can be added to further evaluate the classification effect of the CNN model. In addition, since the control group included in this study was healthy volunteers without stomach disease, there may be some limitations in exploring clinical applications. The exclusion of gastric problem could be to avoid some undiagnosed cancer being included in the control group, but it potentially introduced an opposite bias. In order to make the differential diagnosis of GC more reliable in the clinical setting, more clinical samples of gastritis, gastric ulcer, gastric polyp and other benign gastric lesions may be needed to further establish a more complete and reliable detection method.

In fact, the clinical diagnosis of disease is relatively complicated, and it is difficult to confirm the diagnosis with a single inspection result. The purpose of this study is to explore a non-invasive, fast and convenient method to pre-screen high-risk population of GC, and then perform targeted combined diagnosis of gastroscopy biopsy, so as to help accurately identify patients with GC.

## Conclusion

In conclusion, we measured the serum Raman spectra of GC patients and healthy controls, and analyzed the attribution of the major Raman peaks and the metabolic differences in the blood. Then, four mainstream machine learning algorithms, 1D-CNN, RF, SVM and KNN, were employed to develop diagnostic models for spectral data classification. At the same time, the overall accuracy, sensitivity, specificity, PPV, NPV and the ROC curve were used as evaluation indicators to judge the classification performance of these four methods on these spectral data. The results show that the RF classification model has better performance in the overall evaluation, and it is expected to provide a more accurate diagnostic model for the serum Raman spectrum of the disease.

## Data Availability Statement

The raw data supporting the conclusions of this article will be made available by the authors, without undue reservation.

## Ethics Statement

The studies involving human participants were reviewed and approved by the Ethics Committee of the First Affiliated Hospital of Army Medical University. The patients/participants provided their written informed consent to participate in this study.

## Author Contributions

ML, YZ, and WF conceived and designed the research. ML, HH, and GH conducted experiments. ML wrote the manuscript. ML, HH, HT, KX, CY, and XZ collected clinical samples and processed the data. ML and BL analyzed data. YZ and WF supervised the research and elaborate the manuscript. All authors contributed to the article and approved the submitted version.

## Funding

This work was supported by the National Key Research Program of China (2017YFC0909900), the China National Science Foundation (82172374), the Military Logistics Scientific Research Project (AWS17J010).

## Conflict of Interest

The authors declare that the research was conducted in the absence of any commercial or financial relationships that could be construed as a potential conflict of interest.

## Publisher’s Note

All claims expressed in this article are solely those of the authors and do not necessarily represent those of their affiliated organizations, or those of the publisher, the editors and the reviewers. Any product that may be evaluated in this article, or claim that may be made by its manufacturer, is not guaranteed or endorsed by the publisher.

## Supplementary Material

Supplementary Figure 1 The accuracy-epoch curves **(A)** and loss-epoch curves **(B)** of the One-dimensional convolutional neural network (1D-CNN) model.

Supplementary Figure 2 The distribution of prediction probability in different machine learning algorithms. Each light blue box represents the normal probability and the light yellow box indicates the GC probability. The orange line shows the median value of sample distribution, and the green triangle shows the mean value of sample distribution. The whole box reveals the prediction probability range of test samples in 95% confidence intervals. And the maximum and minimum values in the distribution space are displayed at the top and bottom of each box plot, respectively. In particular, the red dots represent outlier samples in every distribution model.
