# Supplementary figures and images for: A Novel and Rapid Serum Detection Technology for Non-Invasive Screening of Gastric Cancer Based on Raman Spectroscopy Combined With Different Machine Learning Methods
Source: Front Oncol. 2021 Sep 27;11:665176. doi: 10.3389/fonc.2021.665176 (PMC8504718; doi:10.3389/fonc.2021.665176)

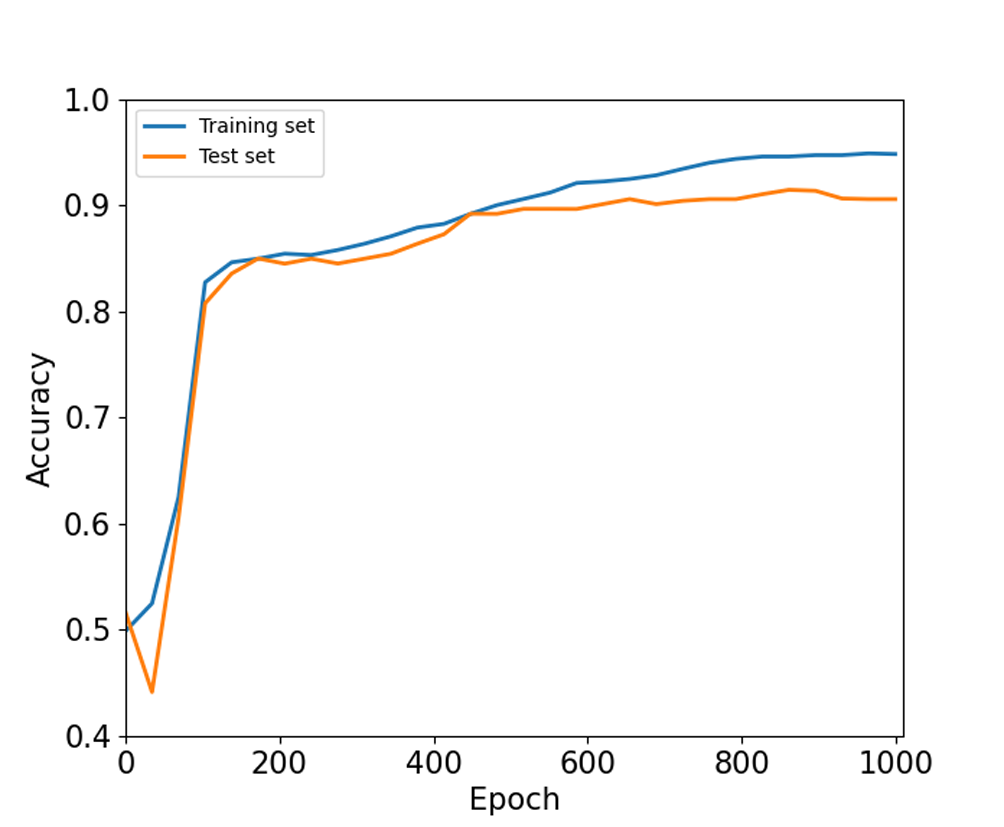

Supplement: Supplementary Figure 1 — The accuracy-epoch curves (A) and loss-epoch curves (B) of the One-dimensional convolutional neural network (1D-CNN) model. [file Image_1.tif]

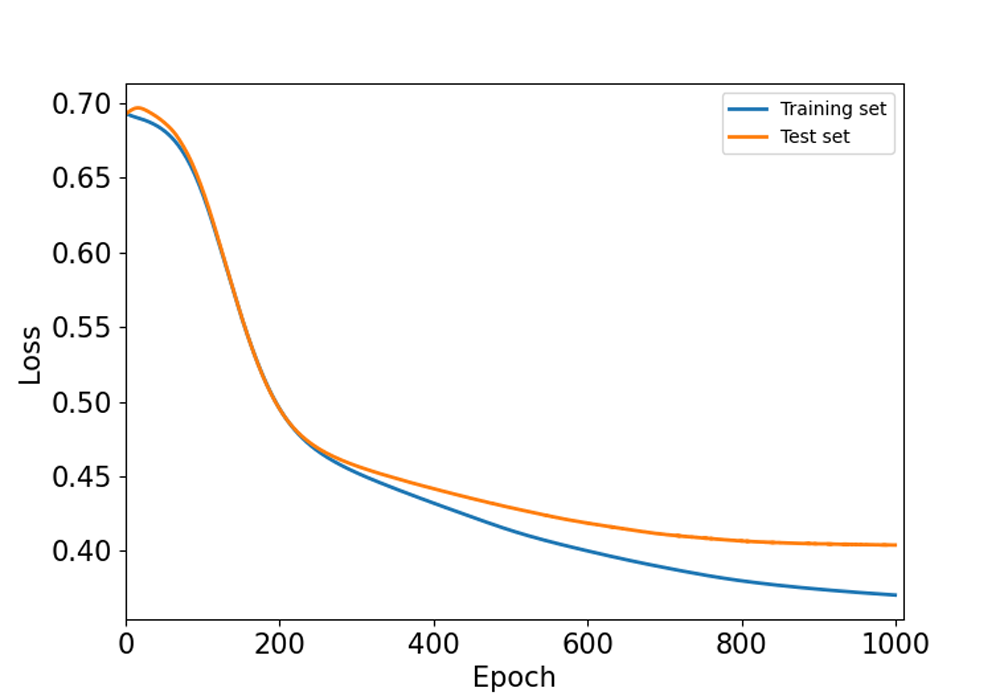

Supplement: Supplementary Figure 2 — The distribution of prediction probability in different machine learning algorithms. Each light blue box represents the normal probability and the light yellow box indicates the GC probability. The orange line shows the median value of sample distribution, and the green triangle shows the mean value of sample distribution. The whole box reveals the prediction probability range of test samples in 95% confidence intervals. And the maximum and minimum values in the distribution space are displayed at the top and bottom of each box plot, respectively. In particular, the red dots represent outlier samples in every distribution model. [file Image_2.tif]

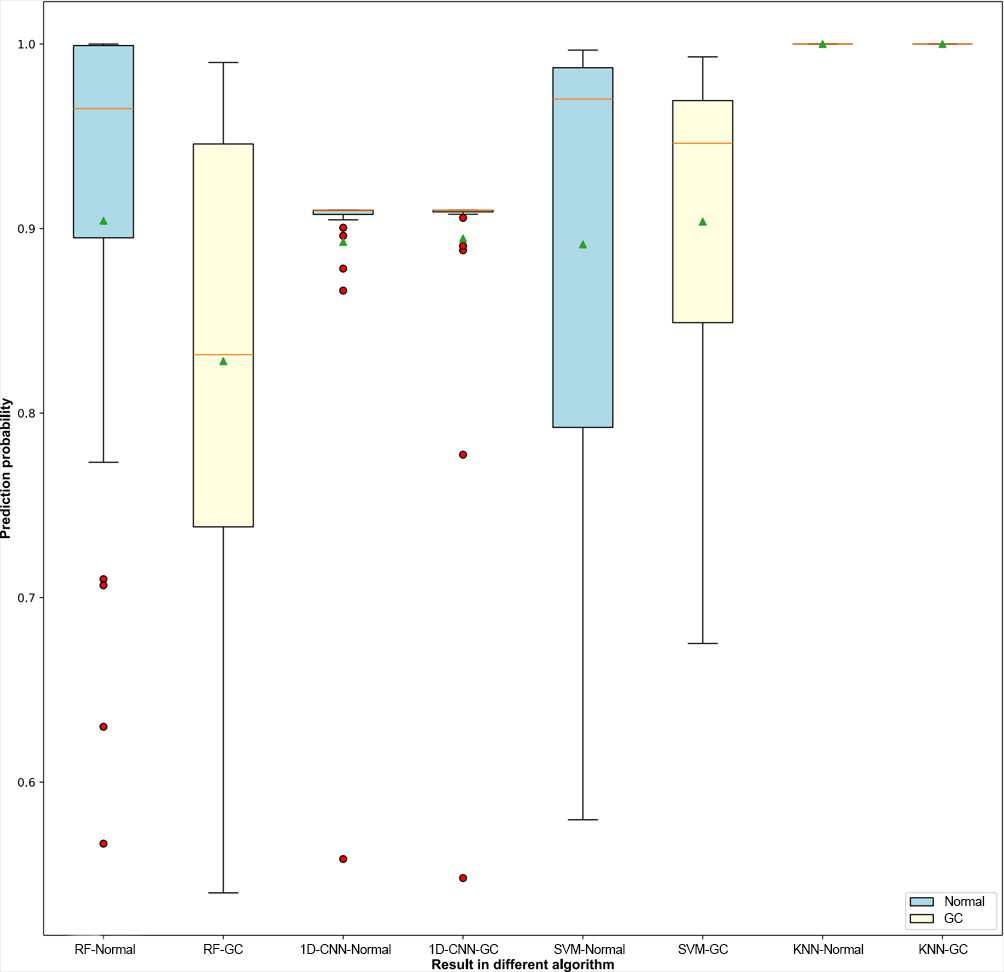

Supplement: Supplementary file 3 [file Image_3.tif]
